# Overview of Cardiac Arrhythmias and Treatment Strategies

**DOI:** 10.3390/ph16060844

**Published:** 2023-06-06

**Authors:** John Kingma, Chantale Simard, Benoît Drolet

**Affiliations:** 1 Department of Medicine, Ferdinand Vandry Pavillon, 1050 Av. de la Médecine, Québec City, QC G1V 0A6, Canada; 2 Faculty of Pharmacy Ferdinand Vandry Pavillon, 1050 Av. de la Médecine, Québec City, QC G1V 0A6, Canada; chantale.simard@pha.ulaval.ca (C.S.); benoit.drolet@pha.ulaval.ca (B.D.); 3 Centre de Recherche de l’Institut Universitaire de Cardiologie et de Pneumologie de Québec-Université Laval 2725 Chemin Sainte-Foy, Québec City, QC G1V 4G5, Canada

**Keywords:** cardiac arrhythmias, electrical remodeling, structural remodeling, pathophysiology, pharmacology, non-pharmacologic interventions

## Abstract

Maintenance of normal cardiac rhythm requires coordinated activity of ion channels and transporters that allow well-ordered propagation of electrical impulses across the myocardium. Disruptions in this orderly process provoke cardiac arrhythmias that may be lethal in some patients. Risk of common acquired arrhythmias is increased markedly when structural heart disease caused by myocardial infarction (due to fibrotic scar formation) or left ventricular dysfunction is present. Genetic polymorphisms influence structure or excitability of the myocardial substrate, which increases vulnerability or risk of arrhythmias in patients. Similarly, genetic polymorphisms of drug-metabolizing enzymes give rise to distinct subgroups within the population that affect specific drug biotransformation reactions. Nonetheless, identification of triggers involved in initiation or maintenance of cardiac arrhythmias remains a major challenge. Herein, we provide an overview of knowledge regarding physiopathology of inherited and acquired cardiac arrhythmias along with a summary of treatments (pharmacologic or non-pharmacologic) used to limit their effect on morbidity and potential mortality. Improved understanding of molecular and cellular aspects of arrhythmogenesis and more epidemiologic studies (for a more accurate portrait of incidence and prevalence) are crucial for development of novel treatments and for management of cardiac arrhythmias and their consequences in patients, as their incidence is increasing worldwide.

## 1. Introduction

The original description of sudden cardiac death in seemingly healthy patients who frequently experience severe fainting spells is attributed to Hippocrates [1]; various electrical diseases contribute to this situation, including long-QT syndrome, arrhythmogenic right ventricular dysplasia or cardiomyopathy (Naxos disease), Brugada syndrome and hypertrophic cardiomyopathy [2]. It was not until the 19th century that Étienne-Jules Marey described the phenomenon of premature ventricular beats along with various aspects of left and right ventricular contraction. Historical aspects of cardiac electrophysiology have recently been discussed [2]. Greater understanding of the physiopathology of cardiac arrhythmias at the subcellular and cellular levels, particularly the distinction between ventricular arrhythmias and atrial fibrillation, is essential for development of novel pharmacologic or non-pharmacologic interventions.

In clinical practice, atrial fibrillation (supraventricular tachyarrhythmia caused by uncoordinated atrial activation and atrial mechanical dysfunction) is a pervasive cardiac arrhythmia, principally brought about by structural and electrophysiological alterations in atrial tissues. It can be encountered in the absence of underlying heart disease but is more frequently seen in connection with mitral valve disease, heart failure, ischemic heart disease and hypertension [3,4]. Early manifestations of atrial fibrillation can be controlled with the use of pharmaceuticals; the overall goal of treatment being to improve survival, reduce incidence of stroke, restore atrial functions, reverse ultrastructural remodeling and improve symptoms.

While ventricular arrhythmias are, for the most part, catastrophic and require immediate attention, atrial fibrillation is usually asymptomatic, undetected and self-terminating. However, over time, atrial fibrillation can evolve to a chronic stage (from paroxysmal), which complicates clinical treatments. In other words, contributory mechanisms as well as triggers change as the disease progresses [5,6]. The principal components that contribute to various stages of atrial fibrillation progression have been characterized [7]. For example, pronounced electrical and structural remodeling along with altered conduction and refractoriness provide the substrate for atrial myocardium to become more vulnerable to reentrant circuit formation [8], shortening of action potential and refractory period duration [9,10,11,12], slowing of conduction and a lower threshold for alternans induction (a crucial element for vulnerable substrate generation [13]. Ultrastructural alterations at the level of the atrial cardiomyocyte membrane probably play a role in pathogenesis of atrial fibrillation but additional structural and functional data are needed. All changes to atrial tissue structure profoundly affect tissue conductivity, wave propagation and potential for reentry.

Clinical and basic science studies have enabled development of clinical strategies to improve quality of life in patients. Herein, we examine experimental and clinical advances that are central to clinical treatment of cardiac arrhythmias. For this review, we searched English-language clinical and basic science reports using the PubMed and Google Scholar platforms with the search terms cardiac arrhythmias, ischemic heart disease, electrophysiology, intrinsic cardiac nervous system, pharmacological treatments, implantable cardioverter defibrillator, catheter ablation, ischemic preconditioning, and combinations thereof.

## 2. General Principles

Cardiac function relies on rhythmic contraction coordinated by specialized cardiac pacemaker cells in mammalian hearts. A well-established electrical conduction system (i.e., sinoatrial and atrioventricular nodes, the His bundle, the right and left bundle branches, the fascicles and the Purkinje fibers [14]) coordinates with cardiomyocytes and other cell types to regulate cardiac function in an orderly fashion. Cardiac action potential requires the highly coordinated action (i.e., opening/closing/inactivation) of plasma membrane ion channel proteins; conduction depends on electrical coupling between different cell types and is mediated by gap junctions [15]. In humans, the cardiac action potential comprises five distinct phases (0–4) (cf. Larson and colleagues [16]): in phase 0, stimulation from the sinoatrial node further brings the membrane potential of atrial myocytes to threshold, thus opening voltage-activated sodium channels—sodium ions diffuse along an electrochemical gradient (from the extracellular space, across the plasma membrane and into the cell). The sodium current produces a positive feedback loop; most of the available sodium currents remain in reserve. Rapid repolarization triggered by fast and slow transient outward potassium currents (phase 1) is followed by a prolonged plateau (due to equilibrium between inward (i.e., L-type calcium, sodium-calcium exchanger) and outward (i.e., potassium—both delayed and inward rectifying) currents (phase 2). The driving force for potassium efflux in this phase is high because of differences between the membrane potential and the potassium Nernst potential [17]. In phase 3, calcium currents are inactivated and outward potassium currents instigate repolarization such that membrane potential is directed towards potassium equilibrium. In phase 4, membrane potential returns to resting values after complete repolarization.

Mammalian cardiomyocytes express a diversity of voltage-gated ion channel pore-forming subunits, which contribute to formation of inward and outward currents that shape waveforms of action potentials and influence both automaticity and refractoriness. The interplay between all of the ionic currents profoundly affects ventricular action potential waveforms. Insights into these relationships have been provided through molecular genetics studies.

In pacemaker cells (distinctive due to properties of automaticity) of the sinoatrial and atrioventricular nodes, atria and the His–Purkinje systems, voltage and calcium dependent mechanisms are involved [18]. Normally, the rate of discharge of the sinoatrial node maintains heart rate between 60–100 beats per minute (bpm). Slower rates of discharge occur in the atrioventricular node (40–60 bpm) or Purkinje system (20–40 bpm); however, these slower rates are normally controlled by the dominant pacemaker, which has a higher intrinsic rate of discharge. Greater automaticity results in a higher rate of action potential discharge due to 1—negative shifts of the threshold potential; 2—a positive shift in maximum diastolic potential; and 3—increased rate of phase 4 depolarization [19]. In the sinoatrial node, this increases heart rate (i.e., sinus tachycardia); the latter is either physiologically caused by greater sympathetic tone or pathophysiologically caused by hypovolemia, ischemia or disturbances in electrolytes. While alternating bradycardia and tachycardia (as seen in patients with tachycardia–bradycardia syndrome) often occurs with atrial fibrillation, the underlying mechanisms remain unclear. Further investigation of different organizational manifestations of phase distribution during arrhythmias could improve characterization of cardiac arrhythmias and provide additional mechanistic strategies for modulating cardiac fibrillation in patients.

In the absence of a vulnerable myocardial substrate and an appropriate trigger, electrical conduction in the heart is reasonably without incident. However, alteration of cellular and electrophysiological properties (i.e., triggered activity, atypical automaticity, re-entry) within the myocardium substantially increases the potential for cardiac arrhythmias. Marked deceleration of early repolarization and acceleration of late repolarization caused by changes in current expression result in atrial fibrillation; molecular changes that may drive these outcomes include 1—augmented inward-rectified potassium current activity, and 2—simultaneous reduction in L-type calcium current activity [5]. Remedial changes include an increase in expression levels of the sodium–calcium exchanger and reductions in rapidly activating outward currents and the fast component of the transient outward potassium current [11,20,21]. The attendant shortened effective refractory period and more negative resting membrane potential increase the window for reentrant excitation [22,23].

Electrical remodeling (i.e., altered functional expression of ionic currents) is associated with ion channel gene mutations but is most important within the context of structural myocardial disease. In the failing heart, electrical remodeling increases susceptibility to both atrial and ventricular arrhythmias [24,25]. Distinct types of ion channel remodeling produce differing types of atrial fibrillation; for instance, tachycardia-induced remodeling involves shortening of atrial refractoriness, while in the aging or failing heart, fibrosis slows conduction velocity, thus prolonging atrial refractoriness. Cardiac wavelength is the physical distance traveled by an electrical impulse within a refractory period; reentry depends on the wavelength being shorter than the total length of the reentrant pathway [26]. A multiplicity of factors, including action potential shortening, induction of depolarizing inward currents and impaired intracellular conduction, contribute to genesis of arrhythmias after acute cardiac ischemia [27]. Acute ischemia increases intracellular resistance and shortens action potential duration consequent to gap junction uncoupling [28]. Persistent cellular uncoupling amplifies spatial differences in repolarization, thereby promoting reentry. Susceptibility to arrhythmias (i.e., ranging from premature ventricular beats to fibrillation) occurs almost immediately after restoration of blood flow to ischemic myocardium [29,30].

Experimental models to evaluate cardiac cell electrophysiology and changes in the time and voltage dependence as well as the time course of ionic currents that underlie the action potential have been available since they were initially reported by Professor Noble in 1962 [31] (cf. in-depth review by Vagos et al. [20]). More recently, in silico (i.e., computational models) drug-screening studies use a mechanistic approach whereby the effects of drug binding are simulated by alteration of gating kinetics of specific ion channels. Using a population models approach [32,33] (as opposed to the single averaged model approach), studies in cardiomyocytes added natural variability to provide more robust and reliable arrhythmia risk markers and metrics [34]. Computational models have also been developed to assess molecular mechanisms that give rise to various aspects of atrial fibrillation, such as spatially discordant alternans [35], which appears to immediately precede atrial fibrillation in patients [13]; contributing mechanisms have not been resolved, but instability in cellular Ca^2+^ cycling may be important [36,37,38].

### 2.1. Genetics

Most cardiac arrhythmias occur as a result of structural myocardial disease, but they also occur in response to various genetic and environmental risk factors and altered epigenetic regulation [39]. The latter are classified by location of origin, polymorphic ventricular tachycardia dominated by primary hereditary arrhythmia syndrome and ventricular fibrillation [40,41].

Increasing interest in determining genes that are responsible for causing hereditary arrhythmogenesis is emerging (cf. recent state-of-the-art review by Wang and Tu [42]); numerous mutations of ion channels that configure the cardiac action potential have been determined. Other mutations are also known to reside in gene coding proteins with different biological functions, such as cytoskeletal architecture, calcium handling, sodium transport and cytokine signaling [43,44,45]. Defects in a host of cytoskeleton proteins (desmin, lamin, titin, filamin, etc.) markedly affect structural integrity and mechanotransduction in cardiomyocytes (cf. review paper by Austin and colleagues [46]). Similarly, mutations in genes that regulate calcium homeostasis (i.e., phospholamban, SERCA2, ryanodine receptor, etc.) can potentially provoke arrhythmogenesis. Genome-wide association analyses are particularly helpful and have revealed a large number of genetic risk variants (mostly located within intergenic/intronic regions) associated with atrial fibrillation [47].

Multiple microRNAs play a fundamental role in regulating key components (i.e., electrical, structural remodeling) of electrical conduction; impairment of any of these components can lead to development of atrial fibrillation (cf. review article [48]). Long non-coding RNAs may also be involved in modulating fibrosis, ion channel function or energy metabolism [49,50,51]. DNA methylation and histone modification might also link genetic variations with predisposition to atrial fibrillation, but more data are needed. Emerging data intimate that histone deacetylases linked to gene silencing could affect post-transcriptional regulation of specific proteins (i.e., cytoskeletal or conductive) in cardiomyocytes and thereby contribute to atrial fibrillation [52,53,54]. Presently, major gaps exist with regard to knowledge of molecular alterations and pathogenesis of cardiac arrhythmias. Different atrial fibrillation risk variants could also act additively in response to epigenetic factors [48].

Epigenetics (changes in gene expression that occur without changes in DNA sequence [55]) examines potential links between external risk factors and internal genetic machineries along with mechanisms to preserve selected gene activity states [56]. Diverse epigenetic processes, such as expression of non-coding RNA molecules, DNA methylation and histone modification, affect expression of genes that produce significant alterations in cellular structure and function [57]. Epigenetic mechanisms can be acquired or inherited, but their actual role in regulation of atrial fibrillation is unclear.

### 2.2. Myocardial Ischemia

Acute obstruction of a coronary vessel produces profound pathological changes in cardiomyocytes (within the area of no blood flow or anatomic area at risk) due to abrupt stoppage of biochemical and metabolic pathways. Reduced oxygen delivery halts oxidative phosphorylation, depletion of intracellular energy phosphate stores and inhibition of myocyte contractile function. Ischemic heart disease is a major contributor to cardiac arrhythmias, which can be life threatening in patients [58]. Ischemic injury causes structural damage as well as electrical instability (due to excessive sympathoexcitation and attenuation of parasympathetic tone) that predisposes to arrhythmias [29,59]. In addition, membrane potential, speed of depolarization and refractory period within ischemic myocardium and between ischemic and non-ischemic regions are significantly modulated. During acute myocardial infarction, automaticity within the atrioventricular node increases to produce focal atrial tachycardia [60]. Parasystole (i.e., an ectopic pacemaker that discharges at a constant rate and competes with the sinus node—the principal pacemaker of the heart) caused by ischemia or infarction prevents conduction of action potentials to the latent pacemaker; a parasystolic focus occurs when conduction to the ectopic pacemaker and exit conduction are compromised [61,62]. Tissue fibrosis caused by ischemia depresses conduction velocity and is accompanied by directional differences in wave front propagation; unidirectional block, wave break and reentry are commonly associated with patchy fibrosis throughout the myocardium [63,64].

Timely opening of an infarct-related artery is essential for the salvage of viable cardiomyocytes in the anatomic area at risk. While restoration of blood flow to the vascular bed of an infarct-related artery using percutaneous coronary interventions or thrombolytic agents delays necrosis, this may be a mixed blessing as further cardiomyocyte damage may occur to reversibly injured myocytes in the area at risk (i.e., reperfusion injury) [65]. A lethal consequence of reflow after regional myocardial ischemia is the occurrence of reperfusion-induced arrhythmias [30]. Multiple contributory factors, including oxidative stress, calcium influx, altered cellular pH, the opening of a mitochondrial permeability transition pore, etc., are likely responsible [66,67,68]. Reperfusion of the ischemia- or infarct-related coronary vessel(s) restores most parameters; for example, amplitude of depolarization is improved, but synchronicity and refractory period are not [69]. The severity of reperfusion-induced arrhythmias depends on the duration of ischemia. With brief (<3 min) or prolonged (>60 min) ischemia, the occurrence of arrhythmias in animal models is relatively low [29]; between these durations of ischemia, the incidence of malignant reperfusion arrhythmias increases markedly.

Structural/electrical remodeling, altered hemodynamic loads and altered neurohormonal signaling occur during ischemia/reperfusion. They markedly change ion channel function, intracellular ion handling and communication. The type of remodeling is dependent on the strength and duration of the stressor (i.e., electrical—tachycardia; mechanical—volume or pressure overload) [70]. The presence of fibrotic tissue significantly modulates myocardial electrophysiological properties and is a key promoter of arrhythmia progression, due to impaired conduction through the myocardium (i.e., a conduction mismatch between a narrow island of tissue and a larger myocardial mass due to blockage of voltage necessary to activate the distal myocardium [71]), induction of impedance mismatches and conduction discontinuity, along with unidirectional blocks and reentry substrates [72]. Proliferation of fibroblasts, which act as current sources or sinks during excitation in affected myocardium, is linked to abnormal automaticity. Consequently, therapeutic approaches that prevent fibroblast proliferation, secretion and connexin expression could be helpful [73]. Initiation and maintenance of atrial fibrillation is attributed to 1—a single ectopic focus (myocytes located within pulmonary veins) that triggers or maintains atrial tachycardia, fibrillation or electrophysiological remodeling, resulting in continuous atrial fibrillation (i.e., atrial fibrillation begets atrial fibrillation [74]); 2—reentrant circuits that activate atria with fibrillatory conduction, resulting in irregular rhythms; and 3—both ectopic activation and single reentry circuits that initiate multiple wavelets (due to numerous distinct electrical circuits being activated within the atria) [75]. The multiple wavelet reentry hypothesis may be the final common pathway for atrial fibrillation. In the structurally compromised heart, prolongation of action potential duration and alteration of action potential dynamics involve downregulation of repolarizing K^+^ currents (i.e., *I_to_*, *I_Kr_*, *I_Ks_*, *I_K_*_1_) and altered handling of intracellular calcium [24]. An important electrophysiological effect of K^+^ current downregulation is a reduced repolarization reserve; as a result, ventricular myocardium is more susceptible to early afterdepolarizations and functional reentry. Repolarization reserve was first introduced in 1998 [76]; this concept suggests that the complexity of repolarization includes some redundancy, therefore loss of a specific component (i.e., *I_Kr_*) will not ordinarily lead to failure of repolarization (cf. Varro and Baczko [77] for further details).

Altered ventricular mechanics and heightened electrical instability in failing myocardium are triggered by irregularities in intracellular Ca^2+^ handling. Mechanisms such as Ca^2+^-mediated inactivation of L-type Ca^2+^ channels, activation of Ca^2+^-sensitive transporters and the Na^+^-Ca^2+^ exchanger all link intracellular Ca^2+^ homeostasis and ventricular action potentials. Alterations in Na^+^ currents and reduced current density also impact conduction in tissues that are adjacent to non-infarcted myocardium in the non-ischemic risk zone [78,79,80]. The speed of wave-front necrosis (i.e., progression of cellular injury from endocardium to epicardial layers of the myocardial wall of the left ventricle) is significantly affected by location, density and expression of gap junction channel proteins [81,82]; abnormal conduction is due, in part, to loss or redistribution of connexin 43 (i.e., a principal gap junction protein) from the intercalated disk to the lateral cell border in ischemic myocardium [83]. Reduced coupling between normal and affected cardiomyocytes has an effect on action potential duration, predisposing cells to conduction block and reentrant excitation [84,85]. The link between atrial fibrillation, elevated connexin 43 expression and modified anisotropy (i.e., due to altered distribution of gap junctions) has been documented [86,87]. In animal studies, protection against atrial fibrillation is associated with reduced connexin 43 expression levels [88] or altered distribution of the gap junction complex [89].

### 2.3. Inflammation

The contribution of inflammation as a cause of cardiac arrhythmias is largely overlooked; however, its role in the development of atrial fibrillation is increasingly apparent, as evidenced by the observed acute increase in inflammatory proteins [90,91,92] in patients. In fact, cardiac or systemic inflammation occurs normally as part of the body’s non-specific response to injury [93]. In a structurally normal heart, acute inflammation may provoke supraventricular ectopic beats; however, a higher incidence of malignant arrhythmias without sudden cardiac death is observed in patients with febrile illness [94]. In failing hearts, electrophysiological remodeling (i.e., ion channel expression, ionic homeostasis, etc.) contributes to alteration of action potential duration, long-QT syndrome, Torsade de Pointes and atrioventricular block and other repolarization abnormalities that can further impact myocardial instability [95]. Inflammatory cytokines induce various arrhythmogenic syndromes via a host of mechanisms that cause inflammatory channelopathies, altered ion homeostasis or ultrastructural remodeling. The anti-inflammatory actions of statins on atrial end-refractory period (i.e., the interval from depolarization to the recovery of excitability) and atrial fibrillation duration in animal and human studies have been reported [96,97].

An unanticipated emergence of cardiac arrhythmias in patients with COVID-19 has been described in recent studies [98,99,100]; potentially related to inflammatory cytokines (i.e., TNF, IL-1, IL-6, etc.) that have a direct influence on cardiac function and indirect systemic changes [95]. In patients with severe COVID-19, a lower incidence of cardiovascular-related death following treatment with glucocorticoids or IL-6 receptor antagonist treatment has been reported [101].

### 2.4. Diet and Metabolic Disorders

A pathological link between dietary disorders (i.e., dyslipidemia, obesity, Type 2 diabetes, insulin resistance, etc.) and cardiac dysfunction has been suggested. Under normal circumstances, the heart uses free fatty acids as an energy source via a regulated equilibrium between cardiac lipid uptake and oxidation. Metabolic disorders produce a situation where lipid levels exceed storage capacity of adipocytes, which then leads to intracellular accumulation of lipid droplets (with attendant disruption of cardiac function). Myocardial lipid accumulation generally impairs fatty acid metabolism and inflammation and increases vulnerability to sustained or even fatal arrhythmias [102,103,104]; however, excessive accumulation of free fatty acids can also augment inflammation responses [105]. The interplay between free fatty acid toxicity and activation of intracellular signaling pathways leads to ion channel remodeling, with subsequent effects on cardiac electrical activity and abnormal conduction [106,107,108]. Protection afforded by polyunsaturated fatty acids via actions on channel gating and membrane properties, including modulation of cardiac connexins, has been discussed [88,109,110]. These findings support the notion of a relationship between diet, inflammation and arrhythmogenesis.

## 3. Pharmacotherapy

The Vaughan Williams classification system (cf. Table 1) is widely used to classify the plethora of anti-arrhythmic drugs and is based on ionic channel involvement and effects on action potential, sinus node function and atrioventricular conduction (see the recent review by Larson et al. [16]). Class I medications have a wide variety of effects that target blockade of sodium channels; these drugs block the rapid inward sodium current, thereby cardiac depolarization and conduction, along with prolongation of repolarization (via blockade of delayed rectifier K^+^ channels). They also affect action potential and effective refractory period duration and thereby influence automaticity. Class II medications (i.e., beta blockers) act by blunting sympathetic activity, resulting in a reduced rate of the initial depolarization of the action potential, which mitigates automaticity and conduction velocity [111]. The use of beta-blockers for treatment of arrhythmias may reduce the risk of sudden death in patients [112]; however, other studies refute this claim [113,114]. On the other hand, combination therapy, with implantable cardiac defibrillators and beta-blockade medications, provides significant benefit in clinical studies [115,116,117]. Class III medications (i.e., potassium channel blockers) act mostly by blocking the delayed rectifier potassium channel, thereby prolonging repolarization [118,119,120]. Class IV medications (i.e., calcium channel blockers) act primarily at the level of the atrioventricular node by blocking slow inward Ca currents; this results in a prolongation of the effective refractory period while having a minimal effect on cardiomyocytes or the His–Purkinje system [121,122]. Although calcium channel blockers are of limited usefulness for most forms of ventricular tachycardia, they are considered a useful adjunctive therapy for catecholaminergic polymorphic ventricular tachycardia (inherited tachycardia in structurally normal hearts during increased sympathetic activity [123]) and idiopathic left ventricular tachycardia [16]. 

Some pharmaceuticals outside the Vaughan Williams classification system are notable for their anti-arrhythmic actions. For example, ranolazine, primarily used as an antianginal medication with its late sodium channel (I_Na-L_)-blocking effect, also acts in a manner similar to amiodarone (i.e., blockade of inward depolarizing and outward repolarizing currents that affect sodium, potassium and calcium currents to prolong action potential duration) to reduce recurrence of arrhythmias [124,125], in addition to its role in controlling atrial fibrillation. The principal antiarrhythmic actions of ranolazine involve the blockade of peak I_Na_, which reduces excitability and leads to prolongation of the end-refractory period (causing reduced activation of the atria) [126]. However, the role of late I_Na_ inhibition by ranolazine in the management of AF has not been established. Several clinical trials (RAID, MERLIN-TIMI, RESTYLE-HCM) have reported variable findings with respect to risk reduction for arrhythmias with ranolazine [125,127,128].

Another antianginal drug, ivabradine (mixed sodium–potassium current blocker), unlike beta-blockers, reduces heart rate without affecting other aspects of cardiac function, such as inotropy [129]. That being said, further information is necessary since there is a greater relative risk of atrial fibrillation (related to symptomatic bradycardia) in patients [130,131,132]. Adenosine, which acts on specialized conduction tissues in the sinoatrial and atrioventricular nodes, reduces automaticity to effectively limit repetitive and paroxysmal monomorphic ventricular tachycardia (i.e., adenosine-sensitive ventricular tachycardia) [133,134,135]. Digoxin produces both mechanical and electrophysiological effects on the heart via inhibition of Na^+^/K^+^ ATPase (raises intracellular calcium) and enhanced contractility (via increased vagal tone at the level of the atrioventricular node), thereby lowering conduction velocity and increasing the effective refractory period [16]. However, use of digoxin in patients has been curbed (except in advanced heart failure and refractory atrial fibrillation) due to a potential increased risk of arrhythmia-mediated mortality [136,137].

While clinical use of pharmacological agents to limit cardiac arrhythmias is the norm, significant cardiac and extracardiac (i.e., neurohumoral activation) side effects have also been described, the most important being their potential proarrhythmic properties in patients with structural heart disease [138]. While clinical trials examining the efficacy of anti-arrhythmic agents on suppression of arrhythmias have provided important findings, some (Cardiac Arrhythmia Suppression Trials I and II) were unable to demonstrate significant protection in patients with serious arrhythmias and were prematurely terminated [139]. Greater understanding of the underlying mechanisms for pathogenesis of arrhythmias at the molecular, cellular and tissue levels is crucial for development of specific pharmacotherapeutic targets, in combination with, or without, other non-pharmacologic interventions.

## 4. Non-Pharmacologic Interventions

First-order treatment against cardiac arrhythmias in patients is principally accomplished using antiarrhythmic drugs. However, evidence of the overall efficacy of pharmacotherapy is incomplete. This has spurred development of interventions such as implantable electronic pacemakers, cardioverter defibrillators and catheter ablation.

### 4.1. Implantable Devices

**Figure 1:**
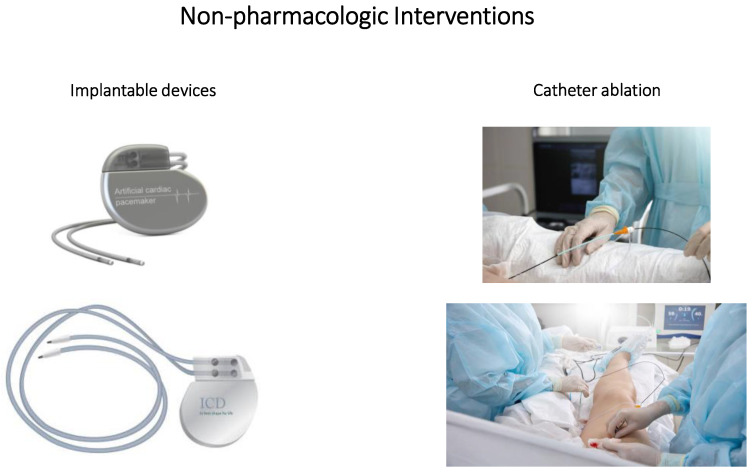
Non-pharmacologic interventions: implantable devices and catheter ablation.

Cardiac pacing (i.e., electrical stimulation to modulate cardiac mechanical activity) was introduced clinically in the 1930s (cf. Figure 1). Electronic pacemaker devices (i.e., unipolar, bipolar) that deliver an electrical pulse sufficient to depolarize myocardium (i.e., stimulation threshold) are standard therapy for symptomatic bradycardia-related symptoms caused by atrioventricular node block or sinus node dysfunction, and severe left ventricular dysfunction [140]. Optimization of parameters such as pulse amplitude and duration is essential to effectively treat symptoms (delayed/absent activation of entire ventricle) in patients [141,142]. While significant advances have been made with this technology, major shortcomings (cost, infection, hemorrhage, lead failure, cardiopulmonary collapse, etc.) have led to a paradigm shift regarding overall use. New battery-less devices are presently under development and should provide benefit for cardiac resynchronization therapy in heart failure patients. Finally, gene-therapy-based manipulation of ionic currents (via delivery of nucleic acid sequences into target cells or tissues), implantation of biological pacemakers that modify cardiomyocytes to provide automaticity or stem cell treatments that add pacemaker syncytia to the heart are presently being investigated [143,144,145,146]. Greater understanding of mechanisms that regulate gene expression and coupling between donor and host cells is essential before biological pacemakers become meaningful therapies for conduction system disorders.

Implantable cardioverter defibrillators were initially used more than 30 years ago in patients recovering from near-fatal ventricular fibrillation and syncope with documented sustained ventricular tachycardia, with or without compromised left ventricular ejection fraction (<40%). Survival, after a two year follow-up period, was significantly greater in these patients compared to pharmacologic treatment [147]. Smaller randomized clinical trials examining the efficacy of these devices post-implant reported that overall survival [148] and risk of adverse outcomes [149] were not enhanced. Although cardioverter defibrillators adequately terminate arrhythmias, they cannot prevent their occurrence [17]. Efficacy of the implantable cardioverter defibrillator requires the capacity to properly detect arrhythmias and to deliver the anti-tachycardia pacing or shock required for cardioversion [150]. Shocks can occur frequently early after implantation and can be painful, thus reducing quality of life (i.e., decreased physical function and mental well-being) for patients [151,152]. Since the publication of these findings, questions have arisen with regard to overall efficacy of implantable cardioverter devices for secondary prevention in patients with multiple comorbidities that have survived a cardiac arrest [153]. Combined treatment with pharmacotherapy in addition to an implantable cardioverter device for management of arrhythmias in patients has recently been shown useful to reduce the defibrillation threshold (i.e., the energy required for defibrillation and restoration of normal sinus rhythm) needed for cardioversion [154]. Future research is warranted to evaluate potential protection using combined pharmacologic and non-pharmacologic treatments for cardiac arrhythmias in patients. 

### 4.2. Catheter Ablation

Retrospective clinical findings suggest that ablation is considerably more effective than conventional pharmacotherapy for treatment of cardiac arrhythmias [155,156]. Recent guidelines suggest that catheter ablation be used for recurrent paroxysmal atrial fibrillation [157], and several clinical trials have documented the superiority of catheter ablation in patients refractory to pharmacotherapy [158,159]. 

Extrinsic (vagus nerve) and intrinsic (network of intracardiac ganglia and interconnecting neurons) components comprise the cardiac autonomic nervous system, which regulates heart rate and cardiac output in response to varying physiologic states [160]. The extrinsic cardiac system contains fibers that connect the heart to the nervous system and the intrinsic cardiac system contains autonomic nerve fibers within the pericardial sac [161]. Cardiac electrophysiology and arrhythmogenesis are significantly impacted by dysfunction of the autonomic nervous system due to the marked diversity in autonomic triggers [162,163]. Identification of these triggers has led to testing of various interventions (i.e., neural ablation or stimulation) that could modulate autonomic activities involved in arrhythmogenesis. Autonomic nervous system dysfunction plays a major role in induction and maintenance of atrial and ventricular arrhythmogenesis [164]; increased sympathetic drive impairs the detrimental effects of ischemia and underlying rhythm disturbances [165]. Catheter ablation of atrial fibrillation is a widely used therapeutic modality in symptomatic patients with irrepressible or persistent atrial fibrillation that are refractory or intolerant to antiarrhythmic drugs. Ablation involves point-by-point lesions (applied using radiofrequency or cryotherapy) that encircle the ipsilateral ostia of the pulmonary veins [166] or other target areas. Ganglionated plexi (i.e., localized neural clusters of intrinsic cardiac ganglia that contain local circuits, parasympathetic neurons and sympathetic afferent and efferent neurons [167]) embedded in adipose tissue on the posterior regions of the atria and the posterior–superior aspect of the ventricles are also targeted [168,169]; however, further validation of ganglionated plexus ablation for treatment of atrial fibrillation is needed. Some collateral damage, including cardiac tamponade, stroke, atrio-esophageal fistula, pulmonary vein stenosis, etc., constitutes a significant drawback to more widespread use of these techniques [166,170]. Nonetheless, considerable immediate and long-term benefit to arrhythmia patients, with regard to recurrence of arrhythmias and quality of life, has been reported with ablation interventions [155,158,171,172,173]. Difficulties associated with this type of intervention include limited resolution of mapping technologies; however, the use of more widely available electroanatomic mapping, magnetic resonance or tomographic cardiac images has helped to improve procedural outcomes [174,175]. A further complication includes the potential for recovery of conduction across previously ablated tissue, which ultimately results in a return of arrhythmias [7]. Controversies surrounding catheter ablation for cardiac arrhythmias have been discussed in a review paper by O’Neill and co-workers [3].

### 4.3. Ischemic Conditioning

Potent, non-pharmacologic protective interventions for lethal ischemia–reperfusion-injury-mediated arrhythmias have emerged in experimental and clinical studies. Ischemic conditioning consists of brief episodes of repeated arterial occlusion/reperfusion carried out prior to a longer episode of arterial occlusion [176] and has been reported to 1—protect against cellular necrosis; 2—preserve post-ischemic cardiac function; and 3—decrease the incidence of cardiac arrhythmias [177,178,179]. Whether antiarrhythmic protection by ischemic conditioning is related to overall protection afforded against tissue injury has not been established.

**Figure 2:**
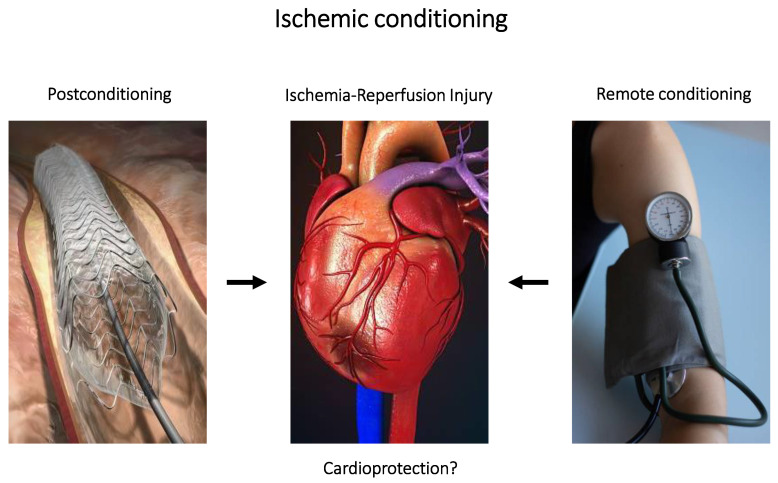
Non-pharmacologic interventions: ischemic conditioning; post-conditioning by angioplasty (**left panel**) and remote (or distant) organ conditioning (**right panel**). The **middle panel** shows site of myocardial ischemia that could be affected by ischemic conditioning treatment.

Despite the many thousands of published studies that report the considerable benefits of ischemic conditioning against ischemic injury, translation to clinical practice remains a particular challenge, in part due to a limited time frame during which potential protective mechanisms can be solicited as well as the random nature of acute myocardial disease (i.e., ischemia, infarction, etc.). Whether ischemic conditioning can abolish or merely delay the onset of ischemia or reperfusion-induced cardiac arrhythmias remains to be established—the same question persists with regard to structural myocardial damage post-ischemia. Nonetheless, the use of post- or remote ischemic conditioning (cf. Figure 2) in patients appears to be promising with regard to arrhythmogenesis since the intervention could be incorporated into current treatment algorithms for primary percutaneous coronary interventions. Theoretically, myocardium subject to ischemic conditioning (after a cardiac event) should provide a substrate where ischemic myocardium is able to more readily adapt to the deleterious effects of re-oxygenation. Spannbauer and colleagues recently conducted a small study in a porcine preparation of acute myocardial infarction where they compared two ischemic conditioning strategies (classic ischemic preconditioning and post-ischemic conditioning) on cardiac arrhythmias [69]; their findings showed that post-ischemic conditioning (i.e., after an ischemic event) exhibited significant antiarrhythmic properties (compared to ischemic preconditioning—carried out prior to an ischemic event). In addition, they reported a significant downregulation of microRNAs (related to cardiac conduction).

A more relevant intervention with clinical potential, remote ischemic conditioning (i.e., brief, repetitive ischemia–reperfusion cycles in an organ or limb distant from the target organ), has been used to limit myocardial injury during cardiac surgery but might also be applied to limit arrhythmogenesis. To date, findings for this intervention in clinical studies remain ambiguous. Significant protection against new onset atrial fibrillation has been reported in patients undergoing cardiac surgery [180,181]; however, other large clinical trials have reported no benefits [182,183,184,185]. A recent meta-analysis of a dozen randomized control clinical studies of more than 5000 patients concluded no benefit to patients with regard to new onset atrial fibrillation [186]; no other types of arrhythmias were investigated. In experimental studies, treatment with remote ischemic conditioning also appears to provide limited protection against ischemia-induced arrhythmias [187,188,189,190]. Differences with regard to anesthetic regimes, ischemic conditioning protocols, animal species, etc., may be partly responsible for the variability in outcomes.

## 5. Perspectives

The prevalence of cardiac arrhythmias and their consequences is increasing worldwide and may be explained by improved diagnostic tools and detection strategies available to clinicians and health care professionals. Worldwide projections are that cardiac arrhythmias affect almost two percent of the global population and are associated with substantial socioeconomic burden. This review article presents recent findings regarding the pathogenesis of cardiac arrhythmias as well as different strategies for their clinical management. Treatment for arrhythmia depends on the underlying cause. Some cases require medication, while others require more invasive procedures such as electrical cardioversion, catheter ablation or implantation of a pacemaker or defibrillator. The ultimate goal of treatment is to restore normal sinus rhythm, so it is imperative that the processes associated with electrical and structural modeling that perpetuate cardiac arrhythmias are reversed. However, it is increasingly clear that pharmaceuticals alone are limited with regard to efficacy for treatment of cardiac arrhythmias. As a result, considerable efforts are underway to find relevant alternatives; combined therapy using pharmacologic and non-pharmacologic interventions should not be excluded.

The identification of risk factors responsible for the pathogenesis of cardiac arrhythmias needs to be addressed. Increasing focus on sex and gender differences is also necessary with regard to the pathogenesis of cardiac arrhythmias [191,192,193]; important electrophysiologic differences between men and women in an aging population have long been described. Present data indicate significant sex and gender differences regarding the incidence and importance of risk factors and etiology of cardiac arrhythmias. Finally, awareness surrounding physiological differences as well as etiology of the differences is un-clear. However, awareness of sex and gender aspects may have important implications for clinical management of all patients with cardiac arrhythmias. What mechanisms, genetic or otherwise, need to be studied to guide future treatment stratagems for primary and secondary prevention of cardiac arrhythmias? Future translational and epidemiologic studies are necessary to help identify novel targets for clinical (and possibly personalized) treatment of cardiac arrhythmias. 

## Figures and Tables

**Table 1:** Vaughan Williams classification of antiarrhythmic drugs.

Class	Drugs
Class I: Sodium Channel Blockers	
Ia	Disopyramide, Procainamide, Quinidine
Ib	Lidocaine, Mexiletine
Ic	Flecainide, Propafenone
Class II: Beta-blockers	Acebutolol, Atenolol, Bisoprolol, Carvedilol, Esmolol, Metoprolol, Nadolol, Propranolol
Class III: Potassium Channel Blockers	Amiodarone, Bretylium, Dofetilide, Dronedarone, Ibutilide, Sotalol, Vernakalant (not available in USA)
Class IV: Calcium Channel Blockers	Diltiazem, Verapamil
Others	Adenosine, Atropine, Digoxin

## Data Availability

No new data were created or analyzed in this study. Data sharing is not applicable to this article.
